# Diamond formation from methane hydrate under the internal conditions of giant icy planets

**DOI:** 10.1038/s41598-021-87638-5

**Published:** 2021-04-14

**Authors:** Hirokazu Kadobayashi, Satoka Ohnishi, Hiroaki Ohfuji, Yoshitaka Yamamoto, Michihiro Muraoka, Suguru Yoshida, Naohisa Hirao, Saori Kawaguchi-Imada, Hisako Hirai

**Affiliations:** 1 National Institute for Materials Science, Tsukuba, Ibaraki 305-0044 Japan; 2 Research and Technology Center, YAZAKI Corporation, Susono, Shizuoka 410-1194 Japan; 3 Geodynamics Research Center, Ehime University, Matsuyama, Ehime 790-8577 Japan; 4 National Institute of Advanced Industrial Science and Technology, Tsukuba, Ibaraki 305-8569 Japan; 5 Japan Synchrotron Radiation Research Institute, Sayo, Hyogo 679-5198 Japan; 6 Faculty of Geo-Environmental Science, Rissho University, Kumagaya, Saitama 360-0194 Japan

**Keywords:** Giant planets, Planetary science, Chemical physics

## Abstract

Hydrocarbon chemistry in the C–O–H system at high pressure and high temperature is important for modelling the internal structure and evolution of giant icy planets, such as Uranus and Neptune, as their interiors are thought to be mainly composed of water and methane. In particular, the formation of diamond from the simplest hydrocarbon, i.e., methane, under the internal conditions of these planets has been discussed for nearly 40 years. Here, we demonstrate the formation of diamond from methane hydrate up to 3800 K and 45 GPa using a CO_2_ laser-heated diamond anvil cell combined with synchrotron X-ray diffraction, Raman spectroscopy, and scanning electron microscopy observations. The results show that the process of dissociation and polymerisation of methane molecules to produce heavier hydrocarbons while releasing hydrogen to ultimately form diamond proceeds at milder temperatures (~ 1600 K) and pressures (13–45 GPa) in the C–O–H system than in the C–H system due to the influence of water. Our findings suggest that diamond formation can also occur in the upper parts of the icy mantles of giant icy planets.

## Introduction

Methane, the simplest hydrocarbon, is ubiquitous in the universe and is one of the key constituents of Uranus and Neptune, along with water and ammonia^1,2^. Experimental and theoretical studies have reported that molecular methane dissociates and polymerises under high-pressure and high-temperature (HPHT) conditions to produce heavier hydrocarbons, while releasing hydrogen to ultimately form diamond^3–9^. The pressure and temperature conditions for diamond formation reported in these studies^3–6^ overlap with the predicted isentropes of Uranus and Neptune^10^. This suggests that diamond produced from methane in the interior of giant icy planets may precipitate and accumulate deep into the planets^1,3,6^. Therefore, knowledge on the hydrocarbon chemical reactions that occur inside carbon-containing outer planets, such as Uranus and Neptune, in the solar system will provide new insights into the mass-radius relationships of these planets and improve models of their internal structure and evolution^10,11^.

The icy mantles of Uranus and Neptune are mostly composed of water and methane, where water may change the conditions of the molecular dissociation of methane^12^. However, experimental studies on the chemical reaction of hydrocarbons under the internal conditions of giant icy planets have been limited to the C–H system with methane, other hydrocarbon species, or polymers (e.g., polystyrene) as starting materials^3–6^. Thus, no HPHT experiments have been performed in the C–O–H system using a mixture of methane and water as a starting material. Furthermore, the conditions for diamond formation in the C–H system reported in previous studies^3–8^ differ substantially among research methods. Theoretical studies^7,8^ with atomistic simulations have predicted that methane decomposes into hydrogen and diamond at pressures of > 300 GPa just above 0 K and > 190 GPa at 2000 K. Likewise, recent dynamic laser compression experiments using polystyrene (C_8_H_8_)_n_ as a starting material showed that HPHT conditions of approximately 150 GPa and 5000 K were required for diamond formation^6^. In contrast, laser-heated diamond anvil cell (LHDAC) experiments using methane as a starting material reported that diamond formation occurred at temperatures above 2000–3000 K in the pressure range of 10–80 GPa^3–5^. These discrepancies may arise from the differences in the kinetics and analytical procedures among the experiments as well as from the assumptions of diamond-formation modelling. In any case, these methods indicate that diamond formation occurs under HPHT conditions; however, how diamond formation proceeds is not fully understood. In particular, there is a lack of knowledge of the process and condition of the chemical reaction that transforms methane to diamond in the C–O–H system. This study reports measurements of the molecular dissociation and polymerisation of methane and diamond formation in the C–O–H system obtained by HPHT experiments using a CO_2_–LHDAC up to 3800 K and 45.0 GPa, with methane hydrate, i.e., a homogeneous water–methane sample at the molecular level, as a starting material.

## Results

**Figure 1:**
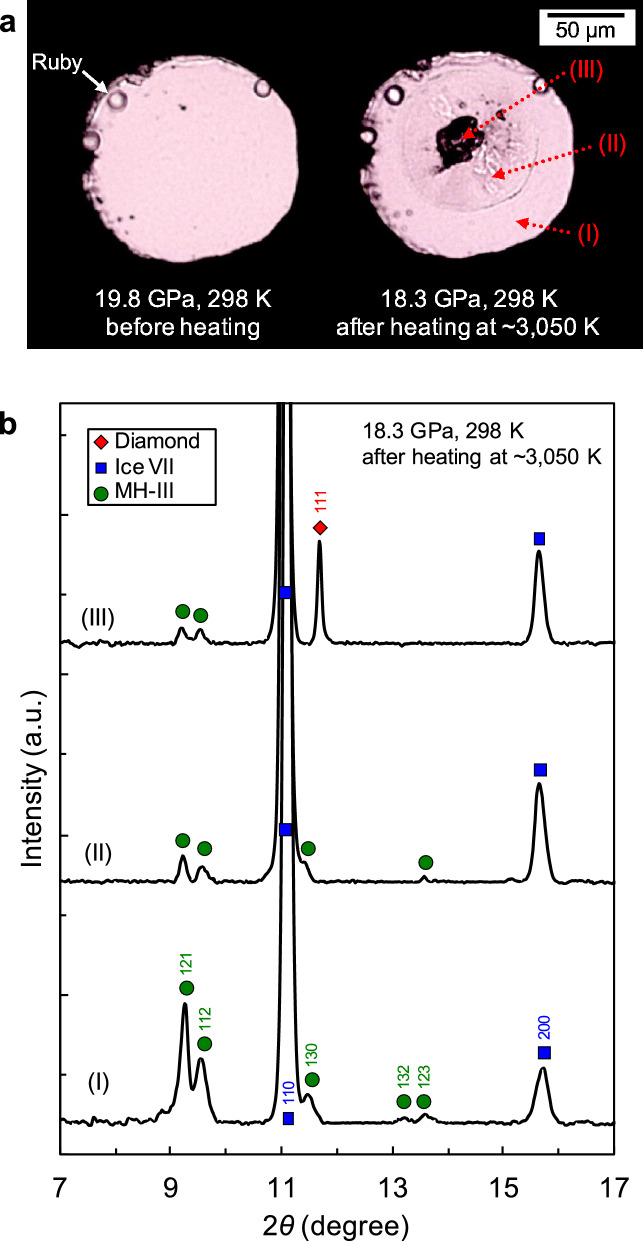
(**a**) Optical micrographs of the sample before and after heating at approximately 3050 K and 19 GPa for 10 min. (**b**) Typical XRD patterns of the heated sample. Each XRD pattern (I to III) was obtained from each measurement point (I–III) indicated by the arrows in (**a**). Green circles, blue squares, and red rhombus show MH-III, ice VII, and diamond, respectively.

Methane hydrate undergoes several phase transitions under high pressure and releases water upon each phase transition^13–16^. Therefore, in our experiments, methane hydrate-III (MH-III) and ice VII coexisted in the sample chamber before heating. Figure 1a shows the visual change in the sample before and after heating. Before heating at 19.8 GPa, the sample was homogeneous with the coexistence of MH-III and ice VII. When the sample was heated to ~ 1200 K, intense convection was observed in the heated area due to sample melting. At a temperature of ~ 1600 K, blackening of the heating centre occurred, and the black region immediately expanded with an increasing temperature of up to ~ 3050 K. After heating at approximately 3050 K and 19 GPa for 10 min, the black area was allowed to cool to room temperature (298 K). To investigate the cause of the blackening of the heating centre, we first performed X-ray powder diffraction (XRD) analyses. Figure 1b shows typical XRD patterns obtained from different measurement points (at the heating centre, the unheated area, and the heated area between these spots) of the heated sample at ~ 3050 K and 19 GPa. In the unheated area, MH-III and ice VII, i.e., as the starting materials, were observed (Fig. 1b(I)). In contrast, at the heating centre, a notable diamond diffraction line was observed in the black area (Fig. 1b(III)). In addition, in the heated area, the diffraction lines of MH-III became significantly weaker, and no other new phases (e.g., graphite) were observed (Fig. 1b(II, III)). One of our previous studies^16^ has shown that MH-III decomposes into solid methane and ice VII at relatively low temperatures (e.g., 633 K at 40.3 GPa). Furthermore, intense convection due to sample melting was observed above ~ 1200 K. Therefore, the weak diffraction lines of MH-III in Fig. 1b(II, III) are considered to be derived from MH-III located near the surface of the diamond anvil away from the laser focusing point, where the temperature did not reach the decomposition temperature of MH-III due to the high thermal conductivity of the diamond anvil, even during heating. These results indicate that the blackening of the sample by heating was due to the formation of diamond, and that diamond formation in the C–O–H system proceeds rapidly.

**Figure 2:**
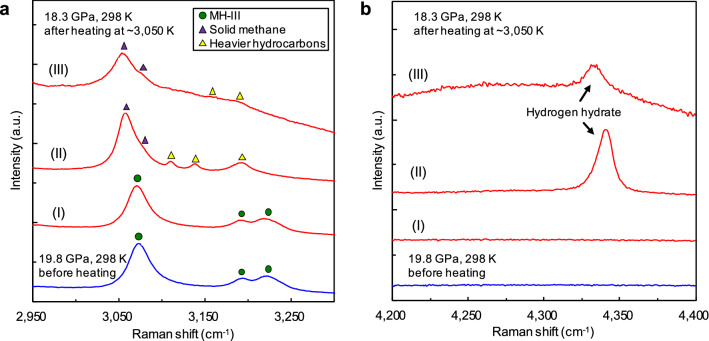
Typical Raman spectra of the sample before and after heating at approximately 3050 K and 19 GPa for 10 min. Each Raman spectrum (I–III) was obtained from each measurement point (I–III) indicated by the arrows in Fig. 1a. (**a**) Formation of solid methane (phase B; purple triangles) and other heavier hydrocarbons (yellow triangles) via the decomposition of methane hydrate (MH-III; green circles) and polymerisation of methane. (**b**) Appearance of the H–H vibration mode of the hydrogen hydrate formed through the reaction between water and released hydrogen via the molecular dissociation of methane.

The source of diamond-forming carbon is the methane component produced by the solid–solid decomposition of methane hydrate^16^; subsequently, diamond formation is the result of the molecular dissociation and polymerisation of methane. When molecular dissociation and polymerisation of methane occur, hydrogen-related materials, such as solid hydrogen derived from released hydrogen, and heavier hydrocarbons different from methane should form in the heating area^4,9^. Thus, to obtain further evidence of the molecular dissociation of methane, we performed Raman spectroscopy in the wavenumber range of the C–H and H–H vibration modes. Figure 2 shows the Raman spectra of the sample before and after heating at approximately 3050 K and 19 GPa. In the unheated area, C–H vibration modes of methane hydrate (MH-III) were observed (Fig. 2a(I)), similar to those before heating. In contrast, in the heated area, not only solid methane (phase B), but also new C–H vibration modes, different from solid methane, were observed (Fig. 2a(II, III)). Although we could not identify new C–H vibration modes, these modes were inferred from previous studies of the C–H system^4,9^, as derived from heavier hydrocarbons, such as ethane, propane, and other long-chain alkanes, which were produced by the polymerisation of methane. At the heated centre of the sample, the Raman intensity of the C–H vibration modes became weaker (Fig. 2a(III)). This suggests that more hydrocarbons were consumed during diamond formation. The formation of diamond in this area was observed in the XRD analyses. The H–H vibration mode of hydrogen-related material was also obtained from the heated area (Fig. 2b(II, III)), which was identified as deriving from hydrogen hydrate based on the relationship between the wavenumber and pressure^17^. These solid products observed by Raman spectroscopy were likely produced from the C–O–H fluid during quenching to room temperature. These products were not detected in the XRD measurements, suggesting that they had low crystallinity, were in an amorphous state, or present in low quantities.

**Figure 3:**
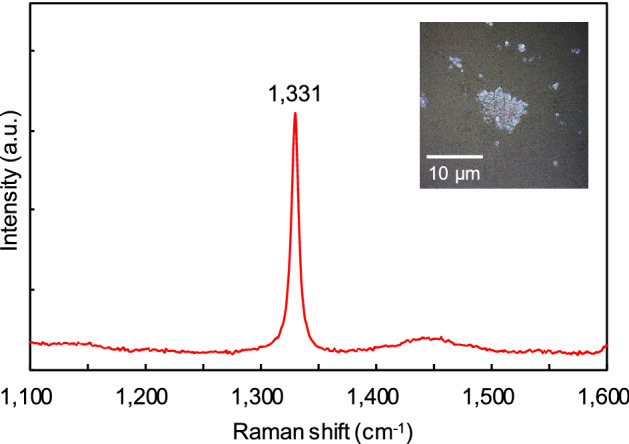
Typical Raman spectrum of the recovered HPHT products after heating at approximately 3000 K and 17 GPa. The spectrum was obtained at ambient condition without DAC.

**Figure 4:**
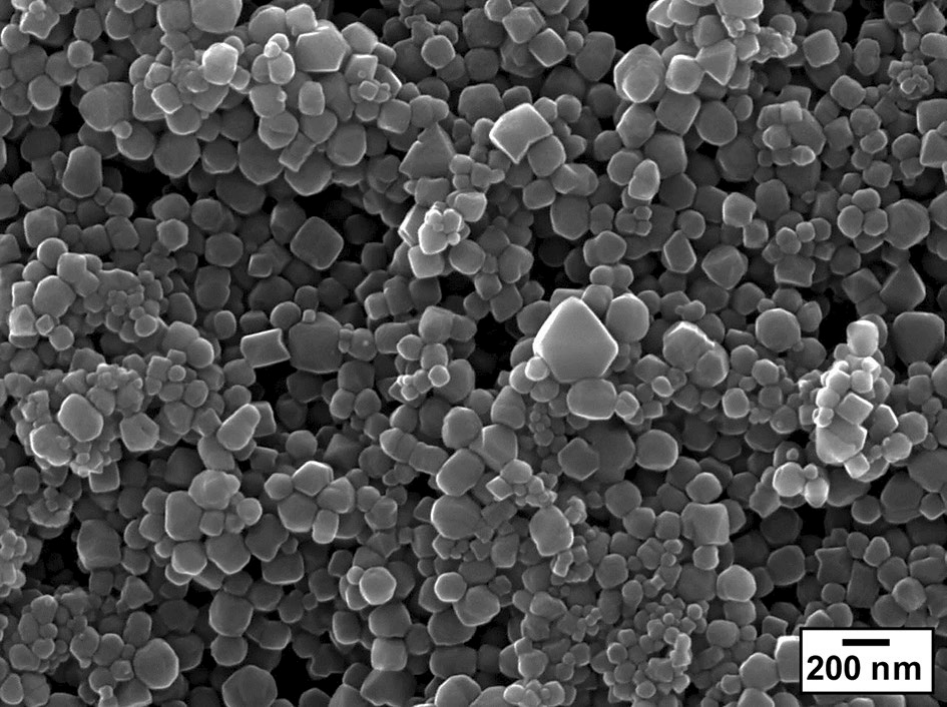
Secondary electron image of the diamond nanoparticles produced from methane hydrate by heating at approximately 3100 K and 40 GPa. These diamonds were recovered at ambient condition after a decompression process.

In this study, Raman spectroscopy and SEM observations were also performed on the recovered sample after the HPHT experiments. Figure 3 shows the Raman spectrum of the HPHT products corresponding to the materials that formed the black area owing to sample heating at approximately 3000 K and 17 GPa. This spectrum was obtained after transferring the HPHT products from the sample chamber onto a glass slide to distinguish the signal of the HPHT products from that of the diamond anvil. The HPHT product that recovered to ambient condition was white with ultrafine particles (inset of Fig. 3), showing the typical Raman peak of diamond at 1331 cm^−1^, whereas we did not observe any signals derived from other materials, such as graphite. Microstructure observations of the recovered HPHT products were carried out using a field-emission scanning electron microscope (FE–SEM). Figure 4 shows the secondary electron image of the recovered sample after heating at approximately 3100 K and 40 GPa, where a majority of crystals with grain sizes ranging from 50 to 350 nm were observed. Some of the particles had an octahedral shape characteristic of diamond; elemental mapping of this recovered product only showed a carbon peak. Similarly, diamond nanoparticles were also obtained from the recovered samples of our experiments carried out at different HPHT conditions (e.g., ~ 3050 K and 19 GPa). The microstructure observations for the recovered samples strongly supported diamond formation in the C–O–H system, as shown by the XRD and Raman spectroscopy analyses. Furthermore, these observations on the recovered samples suggest that the blackening at the heating area of the sample was the result of the agglomeration of diamond nanoparticles formed by the dissociation of methane under HPHT conditions and the scattering of transmitted light at its grain boundaries.

**Figure 5:**
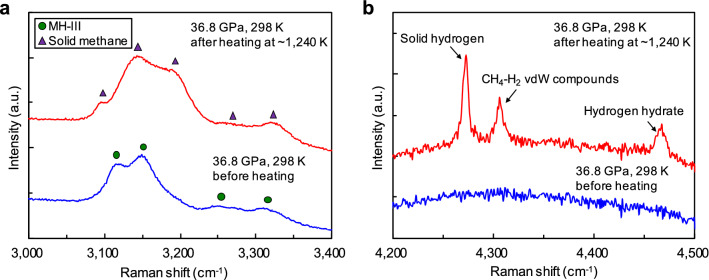
Typical Raman spectra of the sample before and after heating at approximately 1240 K and 37 GPa for 120 min. (**a**) Change in the C–H vibration modes via the decomposition of methane hydrate (MH-III; green circles) into solid methane (phase B; purple triangles) and ice VII. At this pressure, the O–H vibration mode of ice VII shifted out of the measured region^15,16^. (**b**) The H–H vibration modes of hydrogen-related materials produced through the reaction between the released hydrogen via the molecular dissociation of methane and undissociated methane or water, which are abundant in the sample chamber.

The release of hydrogen by the molecular dissociation of methane was observed at temperatures even lower than those of diamond formation. Figure 5 shows the Raman spectra of the sample before and after heating at approximately 1240 K and 37 GPa. Under this condition, no blackening of the heated area occurred and no diffraction line for diamond was obtained by XRD; however, marked changes in the Raman spectra were observed. Before heating, typical C–H vibration modes of MH-III were observed, but they disappeared after heating; instead, typical C–H vibration modes of solid methane (phase B) were observed (Fig. 5a). In addition, the H–H vibration modes of solid hydrogen, CH_4_–H_2_ van der Waals (vdW) compounds, and hydrogen hydrate were observed after heating (Fig. 5b; Supplementary Fig. S1)^17–21^. Moreover, no new C–H vibration mode due to the polymerisation of methane was observed, suggesting that the quantity of heavier hydrocarbons formed under this condition was below the detection limit of our Raman measurements.

## Discussion

**Figure 6:**
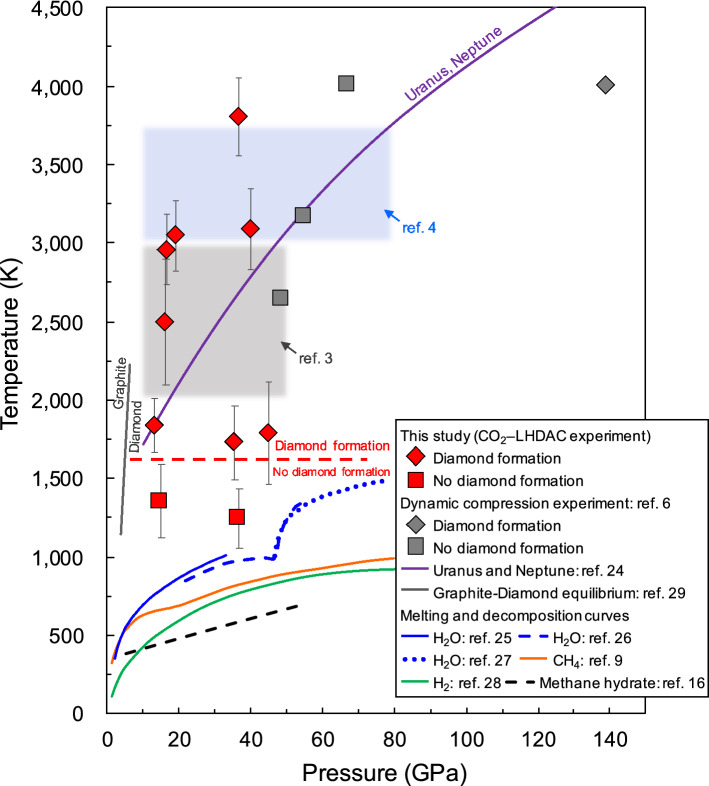
Summary of diamond-formation conditions. The purple solid line is the predicted isentropes of Uranus and Neptune (represented by a single curve owing to the small differences)^24^. Blue, orange, and green lines are the melting curves of water^25–27^, methane^9^, and hydrogen^28^, respectively. The black dashed line is the decomposition curve of methane hydrate^16^. The grey solid line is the equilibrium boundary between graphite and diamond^29^. The red rhombi and squares are the conditions at which diamond formation was observed and not observed, respectively. The diamond-formation conditions in the C–O–H system obtained in this study are milder than those in the C–H system, as reported from atomistic simulations^7,8^, dynamic laser compression experiment (grey rhombus)^6^, and LHDAC experiments (grey- and blue-shaded areas)^3,4^.

This study is the first experimental demonstration of diamond formation in the coexistence of methane and water, and provides more realistic conditions of the interior of the icy mantles of icy planets than previous studies of the C–H system^3–6^. Figure 6 summarises the experimental data on the formation conditions of diamond in the C–O–H system, as shown by our CO_2_–LHDAC experiments with methane hydrate as a starting material. Our results show that the formation of diamond via the molecular dissociation of methane in the C–O–H system occurs at temperatures above ~ 1600 K in the pressure range of 13–45 GPa. The process of diamond formation from methane hydrate under HPHT conditions revealed in this study is as follows. First, as reported in our previous study^16^, MH-III decomposes into solid methane and ice VII at relatively low temperatures (e.g., 633 K at 40.3 GPa) below the melting temperatures of solid methane and ice VII at pressures above ~ 2 GPa. At higher temperatures, solid methane produced by the solid–solid decomposition of MH-III melts, and some of the methane begins to undergo molecular dissociation. For example, above ~ 1200 K, intense convection in the heated area due to the melting of solid methane and ice VII was observed, and solid hydrogen and heavier hydrocarbons formed by the molecular dissociation and polymerisation of methane were observed in the heated samples quenched to room temperature (Supplementary Table SI). It was also found that hydrogen released by the molecular dissociation of methane was fluid during heating, but when it was quenched to room temperature, some of it reacted with water and methane, which were abundant in the sample chamber, to produce various hydrogen-related materials, such as hydrogen hydrate and CH_4_–H_2_ vdW compounds (Fig. 5b; Supplementary Table SI). Above ~ 1600 K, the molecular dissociation of methane is further accelerated, so that methane molecules are completely decomposed into hydrogen and diamond, and this diamond formation caused the blackening of the heating centre of the sample. The blackening of the sample due to diamond formation occurred instantaneously when the temperature reached ~ 1600 K. This indicates that diamond formation in the C–O–H system proceeds rapidly above ~ 1600 K. On the other hand, at temperatures below ~ 1600 K, diamond formation did not occur, even under prolonged heating and pressure conditions (e.g., 120 min at approximately 1240 K and 37 GPa). The diamond formed in the C–O–H system through the above formation process was found to be composed of extremely fine particles with grain sizes ranging from 50 to 350 nm, as revealed by microstructure observations using FE–SEM (Fig. 4).

The obtained temperature for diamond formation in the C–O–H system was > 2400 K lower than that in the C–H system based on dynamic laser compression experiments^6^. Previous studies have also reported that ultra-high pressures of 300 GPa for atomistic simulations^7^ and ~ 150 GPa for dynamic laser compression experiments^6^ are required for diamond formation in the C–H system. In contrast, our results show that diamond formation occurs at substantially lower pressures (i.e., 13–45 GPa). These inconsistencies are thought to be caused by the two major reasons. First, the time scale of studies differs significantly for each method, i.e., picoseconds for atomistic simulations^7^, nanoseconds for dynamic laser compression experiments^6^, and seconds for LHDAC experiments^3–5^. Therefore, the time scales for atomistic simulations and dynamic laser compression experiments are too short to produce diamond from hydrocarbon materials, resulting in the requirements of higher pressures and temperatures. In contrast, our CO_2_–LHDAC experiments, characterised by 10–120 min heating under static conditions, provided a sufficient reaction time for diamond formation via the molecular dissociation of methane; the results revealed the intrinsic conditions for diamond formation in the C–O–H system.

Second, the sample composition in experimental studies differs. The starting materials were polystyrene in dynamic laser compression experiments^6^, methane in previous LHDAC experiments^3–5^, and methane hydrate in this experiment. The amount of diamond produced from carbonaceous materials under HPHT conditions strongly depends on the type of carbonaceous materials used as starting materials^22^. Therefore, in addition to a difference in the timescales of experiments, differences in the hydrocarbon materials used as starting materials may also be responsible for the discrepancy in diamond-formation conditions. Among LHDAC experiments, diamond-formation conditions are not consistent. For example, our CO_2_–LHDAC experiments in the C–O–H system indicated temperatures 400–1400 K lower than those reported by other LHDAC experiments in the C–H system for the production of diamond^3–5^. The main difference between our experiment and previous studies is the presence of water in the starting material; this difference in the sample composition may have a significant effect on the formation conditions of diamond. An experimental study on the synthesis of diamond with stearic acid as a starting material under relatively low pressure and temperature conditions reported that the formation conditions are milder for samples containing volatile fluids, such as water and methane^23^. In addition, a theoretical study conducted in the C–O–H system suggested that ionised water under HPHT conditions acts as a chemical solvent and promotes the molecular dissociation of methane^12^. The results of previous studies^12,23^ support our experimental results.

In conclusion, we clarified the process and conditions of diamond formation from methane hydrate in the C–O–H system for the first time by combining CO_2_–LHDAC experiments with multifaceted evaluations, including XRD, Raman, and SEM analyses/observations. The diamond-formation conditions obtained in this study overlap with the predicted isentropes of Uranus and Neptune (Fig. 6)^24^. Therefore, our results show that the formation of diamond via the molecular dissociation of methane can occur throughout the icy mantles of Uranus and Neptune (even in their upper regions), suggesting that diamond, a dense material produced from methane, settles deep into the icy mantle and accumulates at the boundary between the icy mantle and rocky core of Uranus and Neptune. Our findings provide new insights into the mass-radius relationship of carbon-containing icy planets, such as Uranus and Neptune, which can help improve our understanding of the internal structure and evolution of these planets.

## Methods

The starting material, i.e., methane hydrate phase I (MH-I), was synthesised by the conventional ice-gas interface reaction method^30^ under a fixed pressure and temperature condition of 8 MPa and 269 K at the National Institute of Advanced Industrial Science and Technology (Ibaraki, Japan). The synthesised MH-I sample showed almost full cage occupancy, with a water to methane molar ratio of 5.75–6.05 to 1. When water and methane are loaded in a sample chamber, they usually separate because of the hydrophobic nature of methane. In contrast, methane hydrate can be used as a starting material for a homogeneous water–methane composition at the molecular level because it consists of host cages formed by hydrogen-bonded water molecules and guest methane molecules included in the cage structure^14,31^. Furthermore, by using methane hydrate as a starting material, we were able to simulate the internal conditions of the icy mantles of icy planets with water and methane as their main components more realistically than previous studies of the C–H system^3–9^. We were further able to follow the stepwise chemical evolution of methane to diamond under the coexistence of water. Therefore, methane hydrate was used as the starting material for all experiments in this study. The powdered MH-I sample (average grain size of 2–3 µm) was loaded together with a few ruby balls (approximately 2–5 µm) as pressure markers into a sample chamber in a cryogenic vessel cooled with liquid nitrogen. After loading the sample, the diamond anvil cell (DAC) was warmed to room temperature (298 K) and the sample was pressurised to the target pressure. We used ten methane hydrate samples for the present HPHT experiments (Supplementary Table SI).

Symmetric DACs were used for high-pressure generation. A pair of anvils (300 or 450 μm culet size) was used depending on the target pressure. Rhenium foil with an initial thickness of 250 μm was used as a gasket material after pre-indenting it to a thickness of ~ 50 μm. A CO_2_ laser system at the Geodynamics Research Center of Ehime University was adopted for the heating of the methane hydrate samples. A charge-coupled device (CCD) camera was used for observation in the sample chamber during heating. The laser focal point was approximately 50 μm in diameter, which was sufficiently smaller than the sample chamber. All samples were heated at the Geodynamics Research Center of Ehime University and observed after temperature quenching at high pressure using XRD and Raman spectroscopy. Pressures were determined at room temperature before and after heating using a conventional ruby fluorescence method^32^; the average value of the two pressures was used. The thermal pressure due to laser heating was neglected for simplicity as it was estimated to not exceed 1 GPa^26,27,33^. Temperatures were determined by measuring the thermal radiation spectra emitted from the area of 10–12 μm in diameter at the centre of the heated sample using a spectrometer and fitting it to the grey-body radiation formula. The temperature uncertainty was estimated based on the temperature fluctuation during heating and the fitting error of the emission spectra to the grey-body radiation formula^34,35^. A detailed description of the CO_2_ laser system used in this study has previously been reported^27^.

Synchrotron XRD experiments were carried out for the samples after CO_2_ laser heating using beamline BL10XU at SPring-8, where monochromatic X-rays with a wavelength of 0.04150 nm were used. The beam size was estimated to be ~ 3 μm at the sample position based on the full width at half maximum of the intensity profile of the X-ray beam^36^. A flat panel detector was used to collect XRD patterns, and the exposure time was 100 s. The analysis of the obtained XRD patterns was conducted using the IPAnalyzer and PDIndexer software^37^. Raman spectroscopy was performed using a highly confocal Raman system (PHOTON Design, RSM 800) equipped with a semiconductor laser (λ = 473 nm) at the Geodynamics Research Center of Ehime University. The laser beam was focused at the sample through a × 20 objective lens. The fluorescent signal from the ruby balls in the sample chamber was also collected using the Raman system. The measurement times for the Raman spectrum and ruby fluorescent signal were typically 180 and 5 s, respectively. Peak deconvolution and fitting of all data obtained by Raman spectroscopy were performed using the Fityk software^38^. A field-emission scanning electron microscope (JEOL, JSM-7000F) at the Geodynamics Research Center of Ehime University was used to observe the microstructure of the recovered samples after the HPHT experiments. The acceleration voltage during observations was 15 kV.

## Supplementary Information

Supplementary Information.

## Data Availability

The data that support the findings of this study are available from the corresponding author upon reasonable request.
