# Supplementary material for: Diamond formation from methane hydrate under the internal conditions of giant icy planets
Source: Sci Rep. 2021 Apr 14;11:8165. doi: 10.1038/s41598-021-87638-5 (PMC8047023; doi:10.1038/s41598-021-87638-5)
Supplement: Supplementary file 1 — Supplementary Information. [file 41598_2021_87638_MOESM1_ESM.pdf]

SUPPLEMENTARY INFORMATION

Diamond formation from methane hydrate under the internal conditions of giant icy planets

Hirokazu Kadobayashi[1*], Satoka Ohnishi[2], Hiroaki Ohfuji[3], Yoshitaka Yamamoto[4], Michihiro Muraoka[4], Suguru Yoshida[4], Naohisa Hirao[5], Saori Kawaguchi-Imada[5] & Hisako Hirai[6]

[1] National Institute for Materials Science, Tsukuba, Ibaraki 305-0044, Japan.

[2] Research and Technology Center, YAZAKI Corporation, Susono, Shizuoka 410-1194, Japan.

[3] Geodynamics Research Center, Ehime University, Matsuyama, Ehime 790-8577, Japan.

[4] National Institute of Advanced Industrial Science and Technology, Tsukuba, Ibaraki 305-8569, Japan.

[5] Japan Synchrotron Radiation Research Institute, Sayo, Hyogo 679-5198, Japan.

[6] Faculty of Geo-environmental Science, Rissho University, Kumagaya, Saitama 360-0194, Japan.

*Correspondence to: Hirokazu Kadobayashi

National Institute for Materials Science, Tsukuba, Ibaraki 305-0044, Japan.

E-mail: KADOBAYASHI.Hirokazu@nims.go.jp

Supplementary Figures

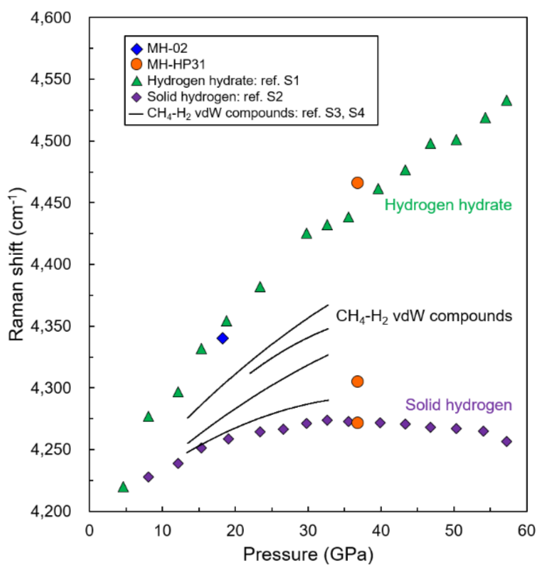

Figure S1. Pressure dependence of the Raman shift of the H-H vibration modes in hydrogen-related materials at room temperature. Green triangles and purple rhombi are changes in the H-H vibration modes of hydrogen hydrate and solid hydrogen with pressure[S1,S2]. Solid black lines are changes in those modes of CH_4-H_2 van der Waals (vdW) compounds with pressure[S3,S4]. Blue rhombus and orange circles are those modes in quenched samples of MH-02 heated at approximately 3,050 K at 19 GPa (Fig. 2b) and MH-HP31 heated at approximately 1,240 K at 37 GPa (Fig. 5b).

Supplementary Tables

Table SI. Experimental conditions and run products observed in the quenched samples.

Run No.	Pressure (GPa)	Temperature (K)	Duration (min)	Reaction products
MH-01	39.9±0.1	3,090±260	10	Dia + HCs + CH_4-H_2 + HH + H_2
MH-02	19.1±0.8	3,050±230	10	Dia + HCs + HH
MH-04	45.0±1.8	1,790±320	30	Dia + HCs + CH_4-H_2 + HH + H_2
MH-05	16.4±0.3	2,500±400	30	Dia + HCs + CH_4-H_2 + HH
MH-08	35.6±0.7	1,730±240	30	Dia + HCs + CH_4-H_2 + HH + H_2
MH-11	13.4±0.2	1,840±170	15	Dia + HCs + HH
MH-R03	36.7±0.1	3,800±250	10	Dia + HCs + CH_4-H_2 + HH + H_2
MH-R06	16.6±1.4	2,960±220	10	Dia + HCs + CH_4-H_2 + HH
MH-12	15.1±0.5	1,350±230	20	HCs + HH
MH-HP31	36.8±0.0	1,240±190	120	CH_4-H_2 + HH + H_2

Dia: diamond, HCs: heavier hydrocarbons, HH: hydrogen hydrate, CH_4-H_2: CH_4-H_2 van der Waals compounds, H_2: solid hydrogen.

Supplementary References

[S1] Machida, S., Hirai, H., Kawamura, T., Yamamoto, Y. & Yagi, T. Raman spectra for hydrogen hydrate under high pressure: Intermolecular interactions in filled ice Ic structure. *J. Phys. Chem. Solids* **71,** 1324–1328 (2010).

[S2] Mao, H. K. & Hemley, R. J. Ultrahigh-pressure transitions in solid hydrogen. *Rev. Mod. Phys.* **66,** 671–692 (1994).

[S3] Somayazulu, M. S., Finger, L. W., Hemley, R. J. & Mao, H. K. High-pressure compounds in methane-hydrogen mixtures. *Science* **271,** 1400–1402 (1996).

[S4] Somayazulu, M. S., Hemley, R. J., Goncharov, A. F., Mao, H. K. & Finger, L. W. High-pressure compounds in the methane-hydrogen system: X-ray, infrared and Raman studies on $CH_4(H_2)_2$. *Eur. J. Solid State Inorg. Chem.* **34,** 705–713 (1997).
